# Supplementary material for: Chalcone-1-Deoxynojirimycin Heterozygote Reduced the Blood Glucose Concentration and Alleviated the Adverse Symptoms and Intestinal Flora Disorder of Diabetes Mellitus Rats
Source: Molecules. 2022 Nov 4;27(21):7583. doi: 10.3390/molecules27217583 (PMC9658082; doi:10.3390/molecules27217583)
Supplement: Supplementary file 1 [file molecules-27-07583-s001.zip › molecules-1994501-supplementary.pdf]

Supplementary Material

Supplementary Tables List:

Tab. S1. Primer sequences for RT-qPCR determination.

Supplementary Figures List:

Fig. S1. Multi samples shannon curves of gut microbiota in the normal rats (A), untreated diabetic rats (B) and diabetic rats treated by high dose DC-5 (10 mg/kg body weight) (C) for 30 days.

Fig. S2. Relative abundance curves of gut microbiota in the normal rats (A), untreated diabetic rats (B) and diabetic rats treated by high dose DC-5 (10 mg/kg body weight) (C) for 30 days.

Fig. S3. Principal component analysis of gut microbiota in the normal rats (A), untreated diabetic rats (B) and diabetic rats treated by high dose DC-5 (10 mg/kg body weight) (C) for 30 days.

Tab. S1. Primer sequences for RT-qPCR determination.

Amplification region	Primer	Sequence
Bacterial 16S V3 + V4	338F	5'- ACTCCTACGGGAGGCAGCA-3'
	806R	5'- GGACTACHVGGGTWTCTAAT-3'
Fungal ITS1	ITS1F	5'-CTTGGTCATTTAGAGGAAGTAA-3'
	ITS2R	5'-GCTGCGTTCTTCATCGATGC-3'
Archaea 16S V3 + V4	Arch349F	5'-GYGCASCAGKCGMGAAW-3'
	Arch806R	5'-GGACTACVSGGGTATCTAAT-3'
Endophytic bacteria	335F	5'-CADACTCCTACGGGAGGC-3'
16S V3 + V4	769R	5'-ATCCTGTTTGMTMCCCVCRC-3'
18S V4	TAReuk454FWD1	5′-CCAGCASCYGCGGTAATTCC-3'
	TAReukREV3	5′-ACTTTCGTTCTTGATYRA-3'
16S full-length	27F_(16S-F)	5′-AGRGTTTGATYNTGGCTCAG-3'
	1492R_(16S-R)	5′-TASGGHTACCTTGTTASGACTT-3'
ITS full-length	ITS1F	5′-CTTGGTCATTTAGAGGAAGTAA-3'
	ITS4	5′-TCCTCCGCTTATTGATATGC-3'
18S full-length	Euk-A_(18S-F)	5′AACCTGGTTGATCCTGCCAGT-3'
	Euk-B_(18S-R)	5′GATCCTTCTGCAGGTTCACCTAC-3'
Bacterial 16S V4 + V5	515F	5'-GTGYCAGCMGCCGCGGTAA-3'
	926R	5'-CCGYCAATTYMTTTRAGTTT-3'
AOA	amoA26F	5'-GACTACATMTTCTAYACWGAYTGGGC-3'
	amoA417R	5'-GGKGTCATRTATGGWGGYAAYGTTGG-3'
AOB	AOB-F	5'-GGGGTTTCTACTGGTGGT-3'
	AOB-R	5'-CCCCTCKGSAAAGCCTTCTTC-3'
nifH	POI F	5'-TGCGAYCCSAARGCBGACTC-3'
	POI R	5'-ATSGCCATCATYTCRCCGGA-3'
nirS	Cd3aF	5'-GTSAACGTSAAGGARACSGG-3'
	R3cdR	5'-GASTTCGGRTGSGTCTTGA-3'
Fungus ITS2	ITS2F	5'-GCATCGATGAAGAACGCAGC-3'
	ITS2R	5'-TCCTCCGCTTATTGATATGC-3'
nosZ	nosRB	5′-CCCGCTGCACACCRCCTTCGA-3'
	nosLB	5′-CGTCGCCSGAGATGTCGATCA-3'
nirK	nirKFLACU	5′-ATCATGGTSCTGCCGCG-3'
	nirKR3CUR3CU	5′-GCCTCGATCAGRTTGTGGTT-3'
narG	narG-F	5′-TAYGTSGGGCAGGARAAACTG-3'
	narG-R	5′-CGTAGAAGAAGCTGGTGCTGTT-3'
Fungus ITS2 (semi	ITS1F	5'-CTTGGTCATTTAGAGGAAGTAA-3'
nested)	ITS4	5'-TCCTCCGCTTATTGATATGC-3'
	fITS7	5'-GTGARTCATCGAATCTTTG-3'
Plant endophytic fungi	ITS5-1737F-ad	5'-GGAAGTAAAAGTCGTAACAAGG-3'
3	ITS2-2043R	5'-GCTGCGTTCTTCATCGATGC-3'

Fig. S1

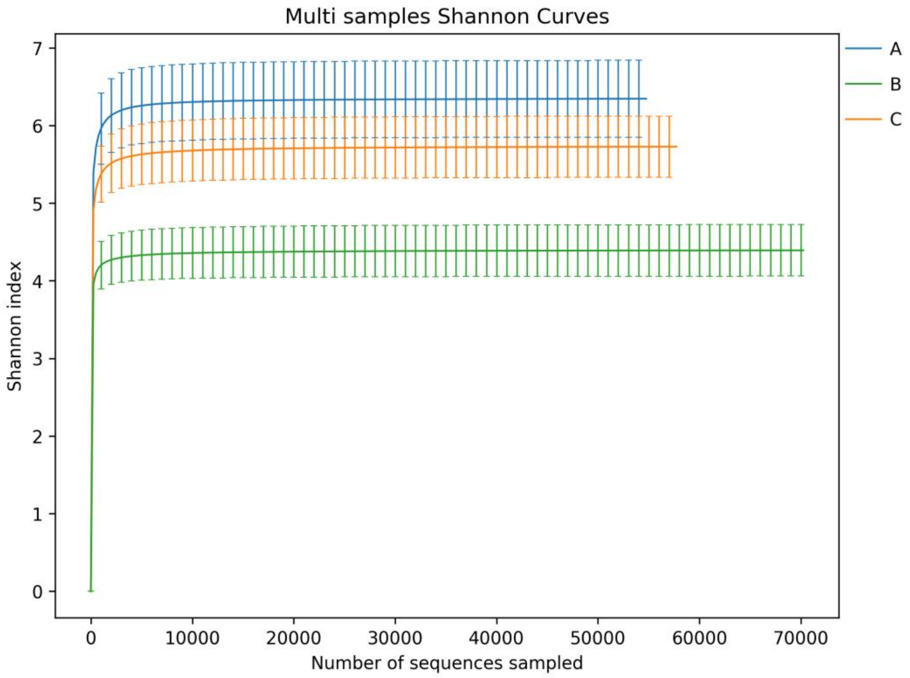
Multi samples Shannon Curves
Shannon index
Number of sequences sampled
0
1
2
3
4
5
6
7
0
10000
20000
30000
40000
50000
60000
70000
A
B
C

Fig. S2

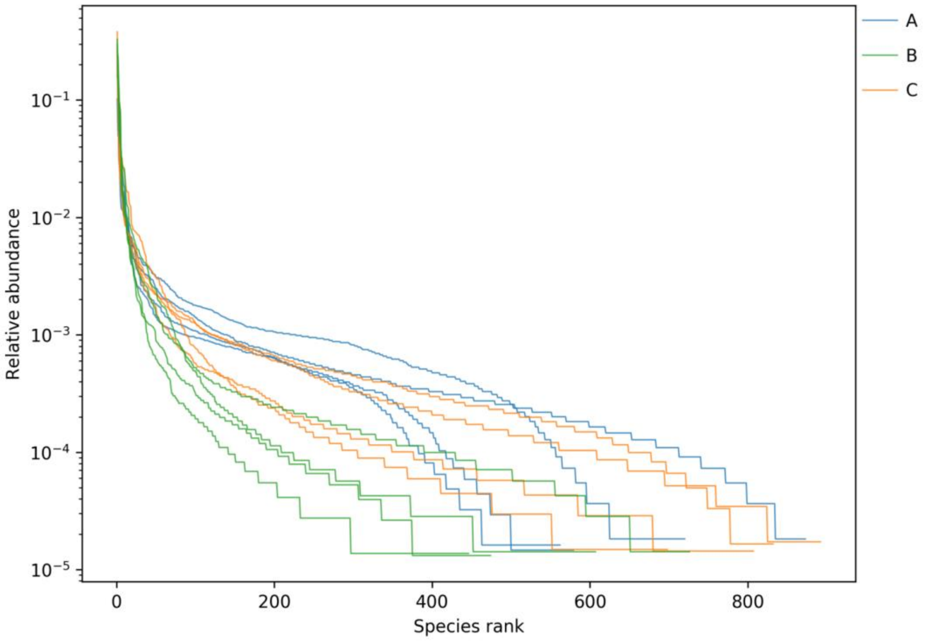
A
B
C
10^{-1}
10^{-2}
10^{-3}
10^{-4}
10^{-5}
Relative abundance
0
200
400
600
800
Species rank

Fig. S3

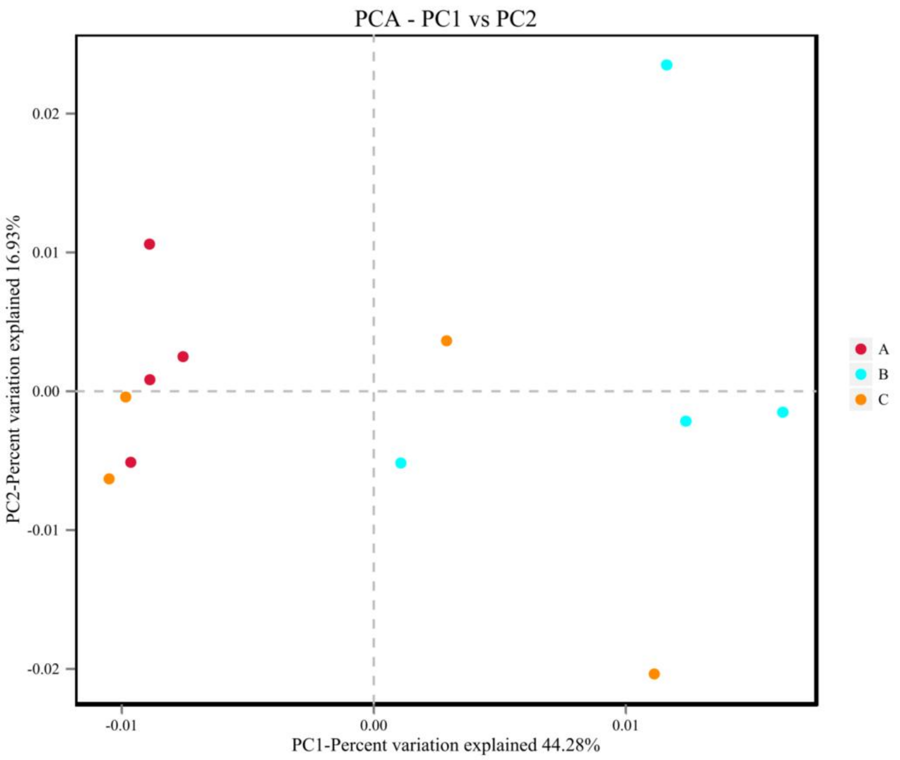
PCA - PC1 vs PC2
0.02
0.01
0.00
-0.01
-0.02
PC2-Percent variation explained 16.93%
-0.01
0.00
0.01
PC1-Percent variation explained 44.28%
A
B
C
